# MolCL-SP: a multimodal contrastive learning framework with non-overlapping substructure perturbations for molecular property prediction

**DOI:** 10.1093/bioinformatics/btaf507

**Published:** 2025-09-11

**Authors:** Yue Luo, Lei Deng

**Affiliations:** School of Computer Science and Engineering, Central South University, Changsha 410083, China; School of Computer Science and Engineering, Central South University, Changsha 410083, China

## Abstract

**Motivation:**

Accurate molecular property prediction remains a central challenge in molecular machine learning, critically dependent on comprehensive molecular representation. Existing methods, however, encounter two major limitations: (i) single-modal learning approaches frequently experience representation bottlenecks, whereas multimodal methods often struggle to effectively leverage complementary information without redundancy across modalities; and (ii) conventional data augmentation techniques typically treat atoms as isolated units, neglecting intrinsic dependencies among atoms within molecular substructures.

**Results:**

Here, we propose MolCL-SP, a substructure-aware multimodal contrastive learning framework specifically designed for molecular property prediction. Our approach integrates molecular representations derived from three complementary modalities using a Transformer-based encoder, followed by modality-specific reconstruction to organically align and fuse cross-modal information. We also introduce a novel substructure-based non-overlapping perturbation strategy for data augmentation, preserving interpretability and effectively enhancing inter-modal interactions. Extensive experimental evaluations demonstrate that MolCL-SP achieves state-of-the-art performance on benchmark datasets for both 2D and 3D molecular property predictions. Additionally, evaluations on drug–drug interaction prediction tasks highlight the model’s strong generalization capabilities. Visualization analyses further indicate that MolCL-SP effectively captures discriminative molecular embeddings even in task-agnostic contexts. Importantly, the model implicitly emphasizes chemically meaningful substructures associated with functional relevance, significantly enhancing interpretability.

**Availability and implementation:**

Codes and materials are available at https://github.com/lylikeeMoon/MolCL-SP.

## 1 Introduction

Molecular property prediction is crucial in computational drug discovery and materials design, enabling efficient pre-screening before costly experiments. Central to this task is molecular representation learning (MRL), which extracts meaningful features from limited labeled data. While traditional methods relied on expert-designed descriptors such as ECFP (Rogers and Hahn 2010), recent deep learning approaches learn continuous, task-specific embeddings directly from molecular inputs, effectively capturing structural and functional information.

Several end-to-end deep learning methods have emerged. For instance, Xiong *et al.* introduced AttentiveFP (Xiong *et al.* 2020), which exploits 2D molecular structures and hierarchical attention mechanisms to generate discriminative molecular embeddings. Schütt *et al.* (2017) proposed SchNet, a model capturing 3D molecular geometries through continuous-filter convolutional networks, effectively encoding spatial and electronic molecular environments.

Despite these advances, acquiring molecular property labels remains costly and time-consuming due to experimental constraints. This leads to significant data scarcity, limiting the effectiveness of end-to-end learning and increasing the risks of overfitting and poor generalization. Self-supervised learning and pretraining-fine-tuning pipelines have been proposed as solutions to these challenges. Self-supervised learning utilizes large-scale unlabeled molecular datasets to generate robust representations, thereby reducing dependency on scarce labeled data and improving model generalizability. Representative examples include SMILES-BERT (Wang *et al.* 2019), which employs masked language modeling and contrastive learning on SMILES strings; MolCLR (Wang *et al.* 2022a), which pretrains graph neural networks via atom masking, bond deletion, and subgraph removal; and 3D InfoMax (Stärk *et al.* 2022), which leverages contrastive learning on local and global molecular geometric features. Nevertheless, these methods often inadequately capture crucial chemical functional groups and structural features, and their augmentation techniques may disrupt meaningful chemical semantics. Thus, developing interpretable MRL frameworks remains crucial for ensuring that learned embeddings are chemically meaningful and robust (Wang *et al.* 2022).

While multimodal pretraining can integrate complementary information from 1D (SMILES), 2D (molecular graphs), and 3D (spatial conformations), existing frameworks still exhibit important limitations. For instance, GraphMVP (Liu *et al.* 2021) and DVMP (Zhu *et al.* 2023) align 2D and 3D modalities only at the whole-molecule level, without explicitly addressing redundant cross-modal information, which can weaken complementarity. Similarly, MOLEBLEND (Yu *et al.* 2024) applies global “modality blending” but does not incorporate substructure-level perturbations or non-overlapping augmentation strategies. These gaps motivate the development of MolCL-SP, which introduces chemically interpretable, non-overlapping substructure perturbations consistently aligned across all three modalities, thereby maximizing complementarity, reducing redundancy, and improving interpretability.

To address existing limitations, we propose MolCL-SP, a multimodal molecular property prediction framework that uses non-overlapping substructure perturbations and contrastive learning. It enhances interpretability by focusing on intrinsic molecular features and leverages complementary information across 1D, 2D, and 3D modalities. MolCL-SP integrates interpretable 1D interpretable substructure-partitioned fingerprints (ESPF) processed by a Transformer, molecular graphs encoded by GIN (Xu *et al.* 2018), and 3D geometries encoded by DimeNet (Gasteiger *et al.* 2020), with modality-specific decoders optimizing reconstruction losses.

We introduce a substructure-aware, non-overlapping perturbation strategy for data augmentation that aligns chemical substructures across modalities at the atomic level. This ensures chemically coherent perturbations that preserve interpretability and effectively capture substructure-level insights through contrastive learning.

Extensive experiments show MolCL-SP achieves state-of-the-art results on key 2D and 3D molecular benchmarks. Feature visualization, interpretability analyses, and successful applications in drug-related association prediction further demonstrate its strong generalization and interpretability.

## 2 Materials and methods

### 2.1 Dataset for pretraining and fine-tuning

For pretraining, we used the PCQM4Mv2 dataset from the OGB large-scale benchmark (Hu *et al.* 2021), which contains 3.37 million molecules with available SMILES, 2D graphs, and 3D geometries. During fine-tuning, we conducted extensive experiments on 20 downstream molecular property prediction tasks across multiple data formats. We evaluated model performance on 8 downstream datasets from MolecularNet (Wu *et al.* 2018) (2D) using ROC-AUC, and on 12 quantum property prediction tasks from the QM9 dataset (Ramakrishnan *et al.* 2014) (3D) using root mean absolute error (MAE). For more details on these datasets, see Section 1.1.2 of the Supplementary Materials. Notably, following previous work (Xia *et al.* 2023a), we adopted scaffold splitting to divide each dataset into training, validation, and test sets with an 8:1:1 ratio. To ensure consistency and avoid potential data leakage, all baseline models were pretrained and fine-tuned using identical scaffold splits. This means that the scaffold-based partitions used during pretraining strictly match those applied during fine-tuning and evaluation.

### 2.2 Overview of Mol-SP

**Figure 1.**
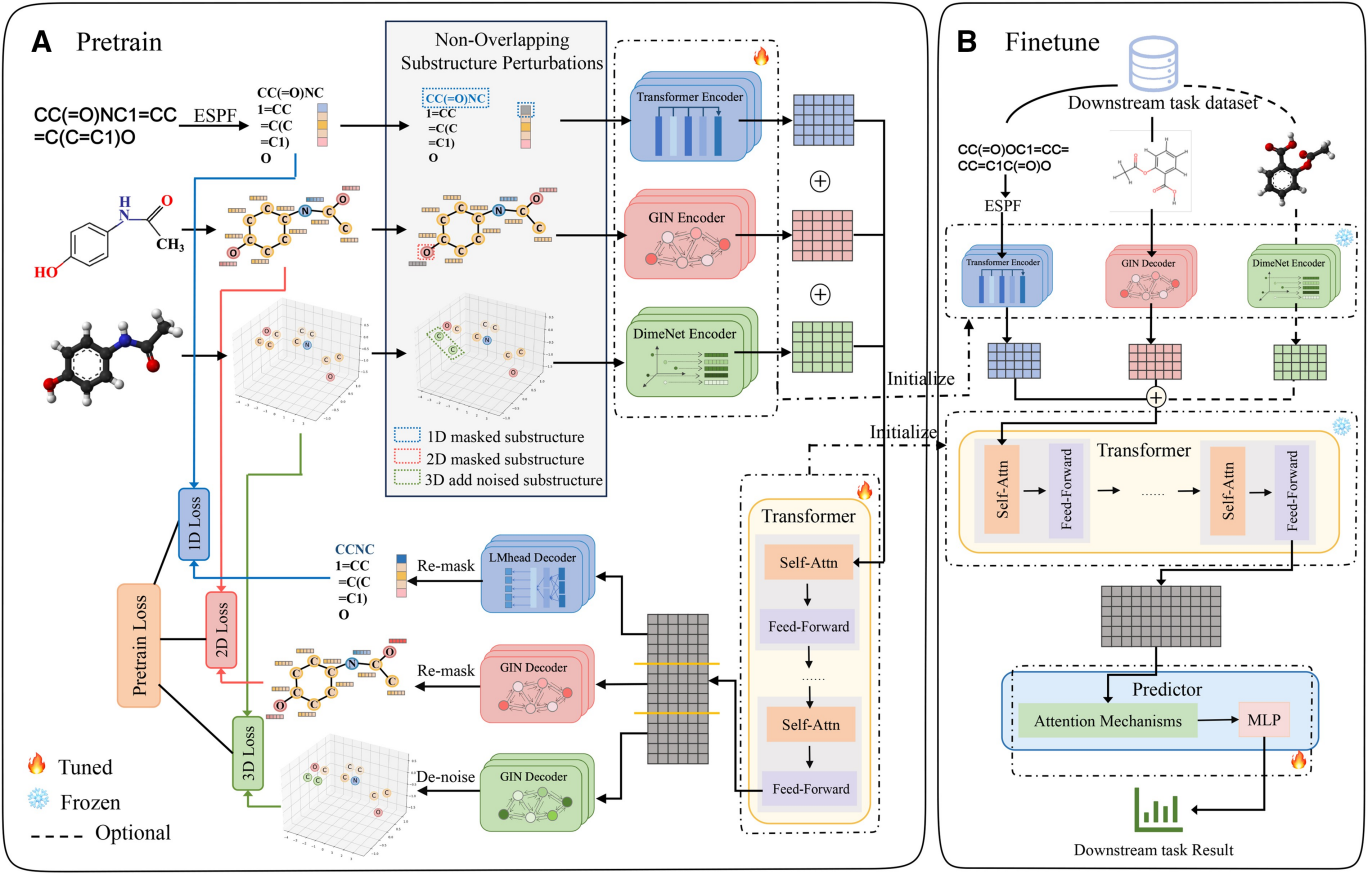
MolCL-SP framework. (A) In pretraining, 1D SMILES, 2D graphs, and 3D geometries undergo substructure-aware non-overlapping perturbations, are separately encoded, and fused via a Transformer, with decoders reconstructing perturbed data for robust representation learning. (B) In fine-tuning, pretrained encoders and Transformer are initialized, modality embeddings are fused with attention, and an MLP predicts molecular properties.

As shown in Fig. 1, Mol-SP mainly consists of two parts: pretraining and fine-tuning. In the pretraining phase, our framework integrates multimodal molecular information by encoding perturbed 1D ESPF sequences, 2D graphs, and 3D geometries using modality-specific encoders, followed by feature interaction via a shared Transformer. The non-overlapping substructure perturbation strategy introduces localized information loss across modalities, generating contrastive views while minimizing redundant perturbations. The fused representation is then split and fed into three modality-specific decoders, with reconstruction losses guiding the learning of robust and interpretable embeddings. Moving to the transfer learning stage, the pretrained encoders and Transformer are reused to process downstream molecular data. Encoded 1D, 2D, and optional 3D features are jointly fused and dynamically weighted through an attention mechanism, and the final representation is passed to an MLP for molecular property prediction.

**Figure 2.**
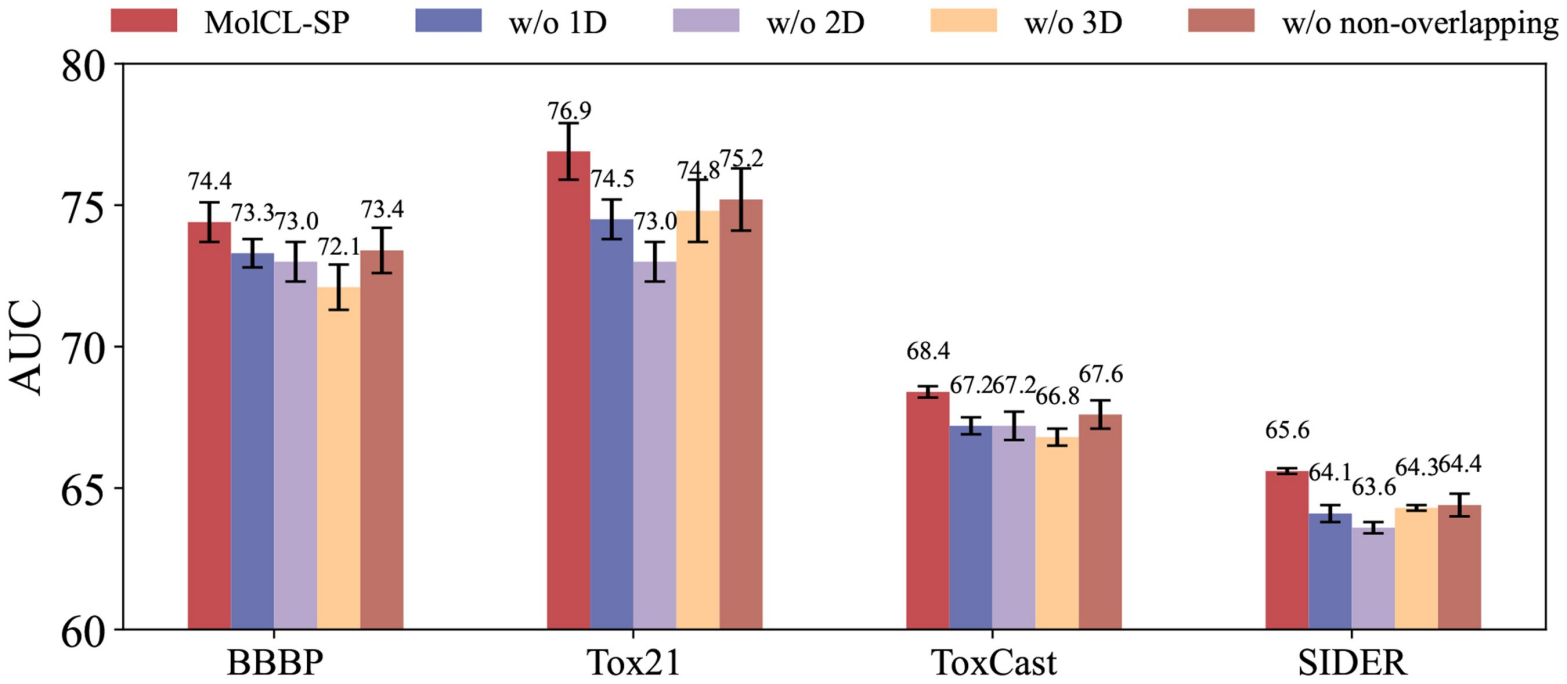
Results of ablation studies on four downstream datasets in MoleculeNet.

#### 2.2.1 Non-overlapping substructure perturbations strategy

Prior studies have demonstrated that functional groups and other substructures offer greater interpretability and predictive power for molecular properties than individual atoms. However, excessive mutual information between modalities—while indicating shared content—can negatively impact multimodal learning by reducing information complementarity and even causing model degradation (Shen *et al.* 2024). To address this, we propose a novel non-overlapping substructure perturbation strategy (see Fig. 1, available as supplementary data at *Bioinformatics* online), which preserves interpretability while minimizing cross-modal redundancy. This strategy involves three steps: aligning substructures across modalities, selecting non-overlapping candidates for perturbation, and applying modality-specific perturbations accordingly.

Take the example of a molecule with *n* atoms and *m* bonds, information from three dimensions includes 1D SMILES *S*, 2D molecular graph G=(V,E) and 3D spatial coordinates $C\in R^{n\times 3}$. For 2D molecular graphs *G*, we construct node features $H=\{h_{1},h_{2},\ldots ,h_{n}\}$ and edge features $E=\{e_{1},e_{2},\ldots ,e_{m}\}$ based on atomic and bond properties (details in Supplementary Materials, available as supplementary data at *Bioinformatics* online).

We firstly applied ESPF to SMILES to capture fine-grained local structural features that enhance substructure representation (Vaswani *et al.* 2023). Based on the substructure vocabulary *B*, SMILES are segmented into *e* meaningful SMILES fragments $F=\{f_{1},f_{2},\ldots ,f_{e}\},f_{i}\in B$ and obtained a sequence of token ids $T=\{t_{1},t_{2},\ldots ,t_{e}\}$. Considering that single bonds are often omitted, aligning substructures by matching bond types one-to-one is impractical, so we align substructures by matching atoms and their atom-to-substructure assignments. We first determine the number of substructures to perturb in each modality according to the specified ratios, and then randomly select them in the order of 1D, 2D, and 3D. Substructures selected in lower-dimensional modalities are excluded from higher ones to ensure non-overlapping. Finally, we randomly mask token IDs in the SMILES sequences to obtain $T^{\prime }$, apply feature masking to atoms in the 2D substructures to generate $G^{\prime }$, and introduce Gaussian noise to the atomic coordinates of selected 3D substructures to produce $C^{\prime }$.

#### 2.2.2 Contrastive learning backbone

In our contrastive learning framework, we employ three independent encoders to embed latent information from distinct molecular modalities, projecting them into a shared embedding space for subsequent Transformer processing.

For 1D information, masked token ids $T^{\prime }$ are fed into the 1D encoder Transformer to produce high-dimensional substructure embeddings $T_{m}\in R^{e\times z}$. The Transformer effectively models long-range dependencies among substructures and is well-suited for extracting contextual information from ESPF sequences. More details about the calculation process of the Transformer can be found in the Supplementary Materials, available as supplementary data at *Bioinformatics* online. To incorporate positional information of each substructure, positional embeddings $P_{m}\in R^{e\times z}$ are generated via one-hot encoding followed by another embedding layer, which are summed with the substructure embeddings as the initial input and fed into the 1D encoder to gain 1D embeddings $X_{1D}\in R^{e\times d}$.

Given that graph isomorphism network (GIN) (Xu *et al.* 2018) can distinguish between most non-isomorphic graph structures—critical for capturing subtle topological variations (e.g. functional group positioning)—we employ it as our 2D encoder. The masked molecular graphs $G^{\prime }$ are encoded by GIN into a shared d-dimensional feature space to obtain 2D embeddings $X_{2D}\in R^{n\times d}$.

For 3D representations, inputs include atomic features *V* and perturbed spatial coordinates $C^{\prime }$. Compared to distance-only models like SchNet (Schütt *et al.* 2017), DimeNet (Gasteiger *et al.* 2020) exhibits superior robustness to conformational changes and explicitly models directional 3D interactions (e.g. hydrogen bonds), making it an outstanding choice as a 3D encoder. It projects perturbed 3D structures into the same d-dimensional space to yield 3D embeddings $X_{3D}\in R^{n\times d}$.

To enable fine-grained alignment and organic integration of atomic representations across each modal, we employ a Transformer-based feature interaction module, a proven superior approach for cross-modal interaction. Before concatenating, the modal-specific trainable parameters $A^{1D}\in R^{e\times d}$, $A^{2D}\in R^{n\times d}$, and $A^{3D}\in R^{n\times d}$ are summed with input features $X_{1D}$, $X_{2D}$ and $X_{3D}$, respectively, to preserve modality identity. They are initialized randomly and updated during training to capture unique characteristics of each modalities. The adjusted feature matrices were concatenated in order to obtain the inputs to the feature interaction module. The feature interaction module consists of stacked identical blocks, each comprising a multi-head self-attention layer and position-wise feed-forward network, with residual connections (He *et al.* 2015) and layer normalization (Ba *et al.* 2016) applied at every layer, ultimately generating the fused embedding output.

The output of feature interaction module are decompose into modality-specific components: ${\tilde{X}}_{1D}\in R^{e\times d}$, ${\tilde{X}}_{2D}\in R^{n\times d}$, and ${\tilde{X}}_{3D}\in R^{n\times d}$ for reconstruction tasks. We design three decoder heads targeting modality-specific objectives. The 1D decoder, implemented as the LMhead of RoBERTa, predicts masked SMILES tokens from ${\tilde{X}}_{1D}$ using a cross-entropy loss (Equation 1). The 2D and 3D decoders are lightweight GIN-based models, reconstructing masked node features and denoising perturbed atomic coordinates from ${\tilde{X}}_{2D}$ and ${\tilde{X}}_{3D}$, respectively. The former is optimized using structural contrastive embedding loss (Equation 2), while the latter integrates L2 loss (mean squared error) (Equation 3) and cosine similarity loss (Equation 4) for refinement (Equation 5). The overall pretraining loss is the sum of the three modality-specific objectives.


(1) $$L_{\text{ce}}^{1D}=-\sum_{i\in M_{1D}}\;\text{log}\;p(t_{i}|{\tilde{1D}}_{m}^{\text{decoded}})$$

(2) $$L_{\text{sce}}^{2D}=\sum_{i\in M_{2D}}{\left(1-\frac{\langle h_{i},h_{i}^{\text{target}}\rangle }{\left||h_{i}||\cdot ||h_{i}^{\text{target}}|\right|}\right)}^{\gamma }$$

(3) $$L_{\text{mse}}=\frac{1}{n}\sum_{i=1}^{n}\left|\right|c_{i}-c_{i}^{\text{target}}|{|}_{2}^{2}$$

(4) $$L_{\;\text{cos}\;}=1-\frac{1}{n}\sum_{i=1}^{n}\frac{c_{i}\cdot c_{i}^{\text{target}}}{\left||c_{i}||||c_{i}^{\text{target}}|\right|}$$

(5) $$L_{\text{denoise}}^{3D}=\lambda_{L2}\cdot L_{\text{mse}}+\lambda_{\;\text{cos}\;}\cdot L_{\;\text{cos}\;},$$

where $M_{1D}$ denotes the set of masked token indices and $t_{i}$ is the ground-truth token at position *i*. $h_{i}$ and $h_{i}^{\text{target}}$ represent the predicted and target node embeddings, while $c_{i}$ and $c_{i}^{\text{target}}$ denote the predicted and ground-truth 3D coordinates of atom *i*. The hyperparameter γ controls the sharpness of the contrastive penalty, typically set to 2 as in prior work.

#### 2.2.3 Fine-tuning

The fine-tuning framework initializes encoder and feature interaction parameters from pretraining and optimizes them using downstream task data. Depending on the task modality, 3D representation encoding and 3D feature participation in interaction can be optionally included during fine-tuning. The resulting embedding matrix, with each row representing a substructure feature, is processed by an attention mechanism that dynamically weights features according to their relevance. Detailed computations are provided in Equations (6) and (7).


(6) $$H_{f}={\left[h_{1},h_{2},\ldots ,h_{k}\right]}^{⊤},\;h_{i}\in R^{d}$$

(7) $$\alpha_{i}=\frac{\;\text{exp}\;\left(w^{⊤}\tanh(h_{i})\right)}{\sum_{j=1}^{k}\;\text{exp}\;\left(w^{⊤}\tanh(h_{j})\right)},\;i=1,\ldots ,k$$

(8) $$z=\sum_{i=1}^{k}\alpha_{i}h_{i},$$

where $H_{f}$ is the embedding matrix of the fine-tuned molecules after feature interactions, *k* is the number of substructures within it. Finally, the attention-weighted embeddings are aggregated into a fused molecular representation, which is passed to a multi-layer perceptron for downstream prediction.

## 3 Results

### 3.1 Evaluate on 2D capability

On the 2D-based MoleculeNet benchmarks, we evaluated MolCL-SP against several state-of-the-art pretraining methods, including MolCLR (Wang *et al.* 2022), GROVER (Rong *et al.* 2020), GraphMVP (Liu *et al.* 2021), 3D InfoMax (Stärk *et al.* 2022), GraphMAE (Hou *et al.* 2023), Mole-BERT (Xia *et al.* 2023b), MoleculeSDE (Liu *et al.* 2023a), and MOLEBLEND (Yu *et al.* 2024). Descriptions of these baseline methods are provided in Section 1.4, available as supplementary data at *Bioinformatics* online. Existing multimodal approaches typically employ separate modality-specific encoders and contrastive learning. All models were pretrained using the PCQM4Mv2 dataset.

Table 1 reports ROC-AUC scores for eight classification tasks. To ensure robust evaluation, we followed established experimental protocols, reporting mean and standard deviation across three random seeds. MolCL-SP outperformed all baselines on six out of eight datasets, achieving second-best results on Tox21 and BACE. Remarkably, despite utilizing only partial molecular information, MolCL-SP consistently showed superior average performance, exceeding MOLEBLEND (the second-best method) by over 2.5 percentage points. Notably, on the ClinTox dataset, MolCL-SP improved ROC-AUC scores by nearly 12 points compared to MOLEBLEND. These results confirm that our pretraining approach facilitates detailed molecular distribution modeling, leading to robust initialization for downstream tasks.

**Table 1.** Performance comparison of MolCL-SP with baselines on 2D classification datasets (ROC-AUC, higher is better), the best result is shown in bold, and the second-best result is underlined.

Methods	BBBP	Tox21	ToxCast	SIDER	ClinTox	MUV	HIV	Bace	Avg
MolCLR^a^	66.6 ± 1.8	73.0 ± 0.1	62.9 ± 0.3	57.5 ± 1.7	86.1 ± 0.9	72.5 ± 2.3	76.3 ± 2.4	71.5 ± 3.1	70.79
GROVER^a^	70.0 ± 0.1	74.3 ± 0.1	65.4 ± 0.4	64.8 ± 0.6	81.2 ± 3.0	67.3 ± 1.6	62.5 ± 0.9	82.6 ± 0.7	71.01
GraphMVP^a^	68.5 ± 0.2	74.5 ± 0.4	62.7 ± 0.1	62.3 ± 1.6	79.0 ± 2.5	75.0 ± 1.4	74.8 ± 1.4	76.8 ± 1.1	71.69
3D InfoMax^a^	69.1 ± 1.0	74.5 ± 0.7	64.4 ± 0.8	60.6 ± 0.7	79.9 ± 3.4	74.4 ± 2.4	76.1 ± 1.3	79.7 ± 1.5	72.34
GraphMAE^a^	72.0 ± 0.6	75.5 ± 0.6	64.1 ± 0.3	60.3 ± 1.1	82.3 ± 1.2	76.3 ± 2.4	77.2 ± 1.0	83.1 ± 0.9	73.85
Mole-BERT^a^	71.9 ± 1.6	76.8 ± 0.5	64.3 ± 0.2	62.8 ± 1.1	78.9 ± 3.0	78.6 ± 1.8	78.2 ± 0.4	80.8 ± 1.4	74.04
MoleculeSDE^a^	71.8 ± 0.7	76.8 ± 0.3	65.0 ± 0.2	60.8 ± 0.3	87.0 ± 0.5	80.9 ± 0.3	78.8 ± 0.9	79.5 ± 2.1	75.07
MOLEBLEND^a^	73.0 ± 0.8	**77.8 ± 0.8**	66.1 ± 0.0	64.9 ± 0.3	87.6 ± 0.7	77.2 ± 2.3	79.0 ± 0.8	**83.7 ± 1.4**	76.16
**MolCL-SP**	**74.4 ± 0.7**	76.9 ± 1.0	**68.4 ± 0.2**	**65.6 ± 0.1**	**99.4 ± 0.2**	**82.2 ± 0.8**	**80.9 ± 0.5**	82.9 ± 1.0	**78.84**

a The results are derived from MOLEBLEND (Yu *et al.* 2024).

### 3.2 Evaluate on 3D capability

We further compared MolCL-SP with advanced methods incorporating 3D information on the QM9 dataset, including Distance Prediction (Liu *et al.* 2023b), 3D InfoGraph (Sun *et al.* 2020), 3D InfoMax (Stärk *et al.* 2022), GraphMVP (Hou *et al.* 2023), MoleculeSDE (Liu *et al.* 2023a), and MOLEBLEND (Yu *et al.* 2024). Following previous works (Yu *et al.* 2024), we partitioned the dataset into training (fine-tuning), validation (10 000 molecules), and test sets (10 831 molecules). MAE served as the evaluation metric, with lower scores indicating superior performance. Table 2 demonstrates that MolCL-SP achieved the best performance among multimodal approaches on 8 out of 12 tasks, consistently surpassing baselines on average. These findings reinforce the predictive strength of MolCL-SP and highlight the benefits of reducing cross-modal information redundancy.

**Table 2.** Performance comparison of MolCL-SP with baselines on 3D QM9 datasets (MAE, lower is better), the best result is shown in bold, and the second-best result is underlined.

Methods	Alpha	Gap	HOMO	LUMO	Mu	Cv	G298	H298	R2	U298	U0	Zpve	Avg
Distance Prediction^a^	0.065	45.87	27.61	23.34	0.031	0.033	14.83	15.81	0.248	15.07	15.01	1.837	13.313
3D InfoGarph^a^	0.062	45.96	29.29	24.60	0.028	0.030	13.93	13.97	**0.133**	13.55	13.47	1.644	13.056
3D InfoMax^a^	0.057	42.09	25.90	21.60	0.028	0.030	13.73	13.62	0.141	13.81	13.30	1.670	12.165
GraphMVP^a^	0.056	41.99	25.75	21.58	0.027	0.029	13.43	13.31	0.136	13.03	13.07	1.609	12.001
MoleculeSDE^a^	0.054	41.77	25.74	21.41	0.026	0.028	13.07	12.05	0.151	12.54	12.04	1.580	11.706
MOLEBLEND^a^	0.060	**34.75**	21.47	**19.23**	0.037	0.031	**12.44**	11.97	0.417	12.02	11.82	1.580	10.485
**MolCL-SP**	**0.052**	35.68	**20.09**	20.77	**0.025**	**0.028**	12.76	**11.37**	0.146	**10.49**	**11.32**	**1.507**	**10.353**

a The results are derived from MOLEBLEND (Yu *et al.* 2024).

### 3.3 Ablation study

To assess the contributions of each modality in MolCL-SP, we performed ablation studies using four variants: “w/o 1D, w/o 2D, w/o 3D, and w/o non-overlapping.” The first three variants excluded one modality during pretraining while maintaining identical fine-tuning procedures. The “w/o non-overlapping” variant disabled our substructure-based perturbation strategy, allowing redundant modality interactions.

The results shown in Fig. 2 reveal that removing any modality or the non-overlapping perturbation strategy markedly degrades performance, with the latter causing the most pronounced drop. Specifically, omitting 1D features leads to the largest decline in sequence-dependent tasks (e.g. HIV, drop of 3.9% AUC), confirming their role in capturing implicit SMILES rules. Excluding 2D features notably impairs topology-sensitive tasks (e.g. BACE, drop of 2.9% AUC), while removing 3D features consistently reduces accuracy even in 2D classification datasets, highlighting the value of spatial geometry. Overall, these findings show that MolCL-SP’s advantage arises from the synergy of multimodal integration and substructure-aware perturbations, rather than any single component.

### 3.4 Visualization on molecular representations

**Figure 3.**
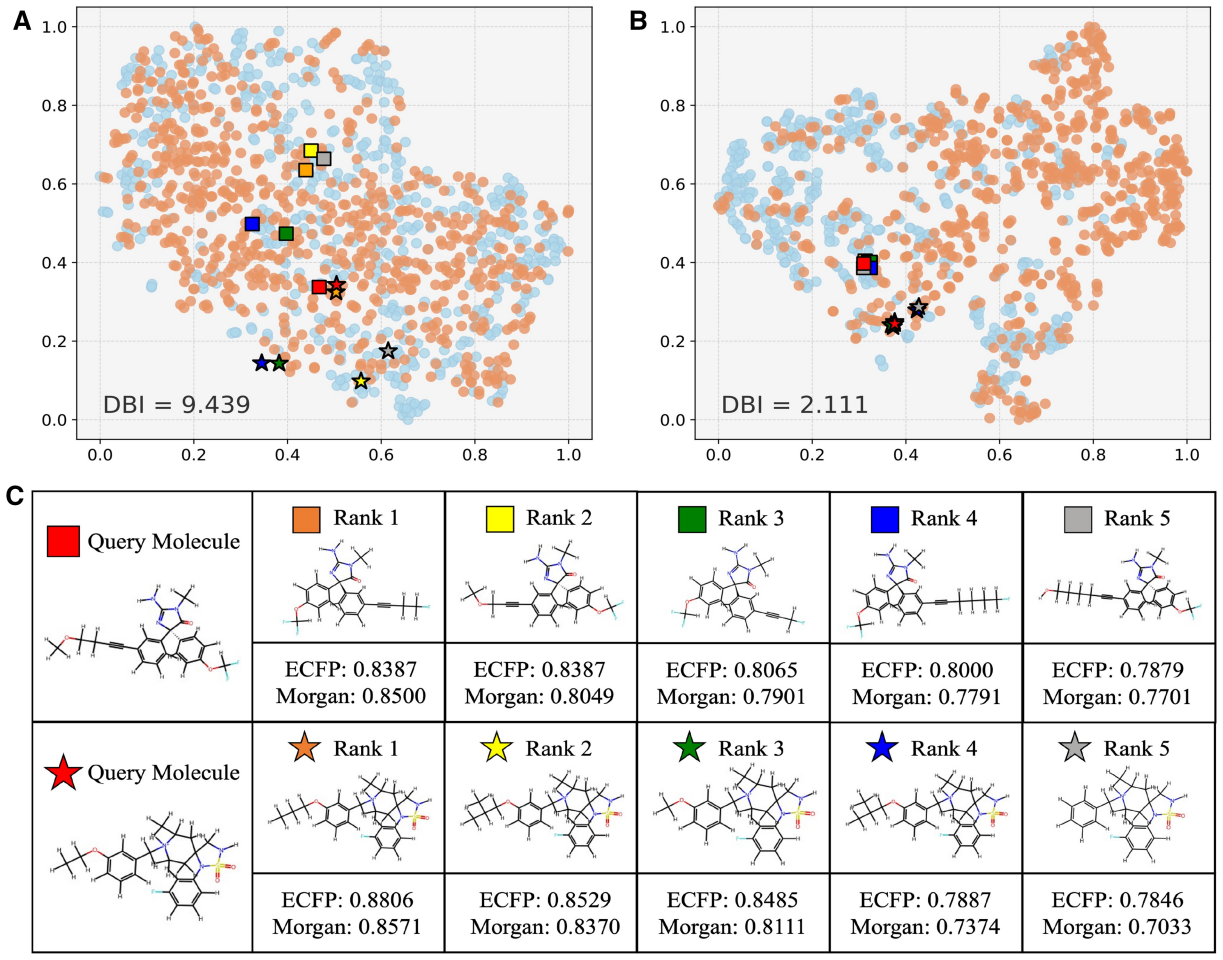
Visualization of molecular representations. (A, B) t-SNE plots of BACE dataset representations extracted by MolCL-SP without and with pretraining. (C) Molecular retrieval results.

To investigate the quality of molecular embeddings learned by MolCL-SP, we applied t-SNE visualization to embeddings from the BACE dataset with and without pretraining. In Fig. 3A and B, positive and negative samples are denoted by blue and orange dots, respectively. MolCL-SP effectively clustered molecules by class, despite no prior exposure to BACE labels during pretraining, yielding significantly improved Davies–Bouldin Index (DBI) scores.

Following Huang *et al.* (2024b), we also conducted molecular retrieval experiments. Randomly selected query molecules from the BACE dataset retrieved structurally similar molecules based on cosine similarity of embeddings. Validation using traditional fingerprints (Morgan and ECFP) Rogers and Hahn (2010) confirmed the high structural relevance of retrieved results (Fig. 3A and B). Pretrained MolCL-SP embeddings demonstrated enhanced discriminative capability, as distant molecules became closer in the feature space. Additional examples are shown in Fig. 2, available as supplementary data at *Bioinformatics* online.

### 3.5 Statistical investigation of interpretability

We further analyzed MolCL-SP’s decision-making through attention weights assigned to functional group-containing substructures during fine-tuning, utilizing a predefined set of 48 functional groups.

**Figure 4.**
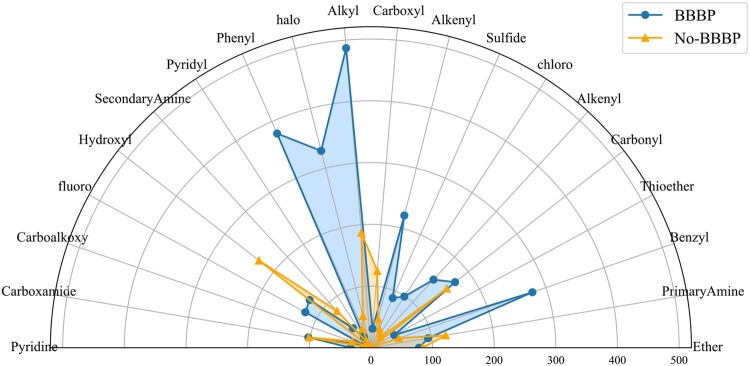
Statistical investigation of interpretability for the functional group prompt: analysis on the BBBP dataset.

According to Zhao *et al.* (2022), lipophilic groups play a crucial role in blood–brain barrier (BBB) permeability, as the BBB’s lipid bilayer favors the diffusion of lipophilic molecules. Additionally, Seelig (2007) highlights that small molecular size also enhances BBB penetration by facilitating diffusion through tight junctions and the lipid bilayer. Thus, molecular weight and lipophilicity are key factors determining BBB permeability. For the BBBP dataset (Fig. 4), permeable molecules show strong attention to hydrophobic groups (e.g. tertiary alkyl, phenyl, benzyl, alkenyl), consistent with known chemistry that these groups enhance BBB penetration. In contrast, non-permeable molecules emphasize hydrophilic groups (e.g. hydroxyl, carboxyl), aligning with their restricted BBB crossing.

For the results from the BACE dataset (see Fig. 3, available as supplementary data at *Bioinformatics* online), active inhibitors received high attention weights on carboxamide, fluoro, and phenyl groups. These substructures are chemically validated to be critical pharmacophores for BACE1 inhibition (Ghosh and Osswald 2014, Müller *et al.* 2023, Scott *et al.* 2016). Incorporating functional group analysis significantly enhances interpretability, guiding more rational drug design strategies.

### 3.6 Model generalizability exploration

To evaluate the utility and generalizability of our molecular representations in drug-related tasks (Deng *et al.* 2020, Xiong *et al.* 2023). Firstly, we applied pretrained MolCL-SP to generate drug embeddings for the drug–drug interaction (DDI) prediction. Following standard protocol (Nyamabo *et al.* 2022), we assessed model performance under both transductive and inductive settings on the Drugbank dataset (Wishart *et al.* 2018). The methods involved in the comparison are embedding extraction method based on Morgan fingerprints (Rogers and Hahn 2010), GMPNN-CS (Nyamabo *et al.* 2022), DGNN-DDI (Ma and Lei, 2023), MSAN (Zhu *et al.* 2022), and SSI-DDI (Nyamabo *et al.* 2021). As summarized in Table 8, available as supplementary data at *Bioinformatics* online, our proposed model MolCL-SP consistently outperforms state-of-the-art baselines across multiple evaluation metrics under transductive settings. Notably, MolCL-SP achieved over 98% on most metrics, and its accuracy exceeded the second-best method by a significant margin of 2.46%. In the inductive setting, as summarized in Table 3, MolCL-SP consistently outperformed all baselines across evaluation metrics under both partition schemes. We believe that MolCL-SP’s strong performance in the inductive setting—where it predicts properties of molecules with no scaffold overlap with the training set—hinges on its substructure-level perturbations, a design choice that fosters learning of transferable local patterns.

**Table 3.** Performance assessment of MolCL-SP and comparison baselines on the DrugBank dataset under the P1 and P2 partitions in the inductive setting, the best result is shown in bold, and the second-best result is underlined.

Method	P1 Partition (new drug, new drug)				P2 Partition (new drug, existing drug)			
	ACC	AUC	AP	F1	ACC	AUC	AP	F1
Morgan	64.17 ± 0.41	72.08 ± 0.40	74.24 ± 1.06	63.73 ± 0.23	75.66 ± 0.58	84.84 ± 0.15	84.87 ± 0.40	78.29 ± 0.16
GMPNN-CS	68.57 ± 0.30	74.96 ± 0.40	75.44 ± 0.50	65.32 ± 0.23	77.72 ± 0.30	84.84 ± 0.15	84.87 ± 0.40	78.29 ± 0.16
DGNN-DDI	70.31 ± 0.62	72.87 ± 0.92	73.65 ± 1.61	67.21 ± 0.59	77.07 ± 0.94	80.35 ± 1.08	82.97 ± 1.45	73.03 ± 0.65
MSAN	66.31 ± 0.61	72.75 ± 0.78	71.61 ± 1.00	68.68 ± 0.60	69.83 ± 1.41	77.29 ± 1.63	75.79 ± 1.95	73.01 ± 0.85
SSI-DDI	65.40 ± 1.30	73.43 ± 1.81	75.03 ± 1.42	54.12 ± 3.46	76.38 ± 0.92	84.23 ± 1.05	84.94 ± 0.76	73.54 ± 1.50
**MolCL-SP**	**74.25** ± **0.21**	**82.56** ± **0.57**	**83.11** ± **0.96**	**73.04** ± **0.47**	**82.57** ± **0.81**	**90.48** ± **1.05**	**90.09** ± **0.93**	**78.84** ± **0.98**

To further evaluate the generalizability of MolCL-SP in the drug-related association prediction, we applied it to identify potential drug–disease association (DDA). We compared MolCL-SP with several state-of-the-art DDA prediction methods, including the deep learning–based DeepDR (Zeng *et al.* 2019), graph convolution–based HDGAT (Huang *et al.* 2024a), as well as multi-view fusion–based AMDGT (Liu *et al.* 2024). The results (summarized in Table 4) show that MolCL-SP is stable and significantly higher than the three comparison methods in three performance evaluation indicators.

**Table 4.** Performance comparison on DDA prediction, the best result is shown in bold, and the second-best result is underlined.

Method	AUC-ROC	AUC-PR	F1-score
DeepDR	0.821 ± 0.013	0.776 ± 0.016	0.783 ± 0.014
HDGAT	0.836 ± 0.011	0.798 ± 0.013	0.801 ± 0.013
AMDGT	0.840 ± 0.010	0.802 ± 0.012	0.805 ± 0.012
MolCL-SP	**0.896** ± **0.007**	**0.863** ± **0.008**	**0.857** ± **0.009**

These results highlight MolCL-SP’s potential to learn transferable molecular representations and to deal with drug-related association prediction tasks.

## 4 Conclusion

MolCL-SP is a multimodal molecular property prediction framework that introduces non-overlapping substructure perturbations into contrastive learning, using molecular substructures as the core unit for augmentation. It integrates 1D, 2D, and 3D modalities via modality-specific encoders and a Transformer-based fusion module, with decoders reconstructing original inputs to enhance robustness. By aligning interpretable substructures across modalities and reducing cross-modal redundancy, MolCL-SP ensures more informative representations.

Extensive experiments show MolCL-SP achieves state-of-the-art results on molecular benchmarks, generalizes well to drug-related prediction tasks, and produces chemically meaningful embeddings, demonstrating its value for computational drug discovery and materials science.

Despite these promising results, opportunities for further improvement remain. Future work may investigate advanced multimodal fusion strategies (e.g. hierarchical attention) to more effectively capture complex cross-modal relationships, incorporate additional biological data such as protein–ligand interactions, and enhance the efficiency of substructure-based pretraining to improve scalability. These directions will further broaden the applicability of MolCL-SP in real-world drug discovery settings.

## Supplementary Material

btaf507_Supplementary_Data

## Data Availability

The data underlying this article are available in zenodo, at https://linkprotect.cudasvc.com/url?a=https%3a%2f%2fdoi.org%2f10.5281%2fzenodo.17367142&c=E,1,SIZj_lT1pvpnxAJZ3y8dsVX98J5PdXmkaSC1Cbtl33kXnnGp7tApzcMy-6JSJgaZeujb7rS1PKB9J-gRguC5jP1Qj-UCCbbC8VVRwltfFA,,&typo=1
