# Supplementary material for: MolCL-SP: a multimodal contrastive learning framework with non-overlapping substructure perturbations for molecular property prediction
Source: Bioinformatics. 2025 Sep 11;41(10):btaf507. doi: 10.1093/bioinformatics/btaf507 (PMC12560823; doi:10.1093/bioinformatics/btaf507)
Supplement: btaf507_Supplementary_Data [file btaf507_supplementary_data.pdf]

MolCL-SP: A Multi-modal Framework for Molecular Property Prediction Based on Non-overlapping Substructure Perturbations and Contrastive Learning

(Supplementary Data)

Yue Luo[1], Lei Deng [1,*]

*To whom correspondence should be addressed.
[1]School of Computer Science and Engineering, Central South University, Changsha, 410083, China

contact: leideng@csu.edu.cn

Contents

1 Details of molecular datasets 2
1.1 pre-training dataset and downstream datasets 2
1.1.1 MoleculeNet dataset 2
1.1.2 QM9 dataset 3
1.2 Input feature construction 4
1.3 Experimental settings 4
1.4 The detail of all baselines. 5
1.5 Calculation process of Transformer, GIN and Dimnet 6
1.5.1 Transformer 6
1.5.2 GIN 6
1.5.3 Dimnet 7
1.6 Non-overlapping substructure perturbations strategy 8
2 Additional experiments and analysis 9
2.1 More visualization findings on molecular representations 9
2.2 Profile of Predefined Functional Groups 10
2.3 Ablation study 12
2.3.1 Module ablation research 12
2.3.2 Component ablation research 12
2.4 Parameters sensitivity analysis 14
2.5 Statistical investigation of interpretability 15
2.6 Performance of MolCL-SP on the drug-drug interaction prediction task.. 16
2.6.1 Drug-drug interaction (DDI) prediction task 16
2.6.2 Drug–disease association (DDA) prediction task 17
2.7 Computational Efficiency Analysis 18

1 Details of molecular datasets

1.1 pre-training dataset and downstream datasets

1.1.1 MoleculeNet dataset

Supplementary Table S1 provides a comprehensive overview of the benchmark datasets utilized in our study, detailing the task types, evaluation metrics, and the number of molecules. These datasets, sourced from MolecularNet, encompass a wide range of molecular properties and tasks, including physiology, biophysics, and other related fields.

Supplementary Table S 1. The description of the benchmark datasets used in our work.

Task type	metric	Category	Dataset	Tasks	Molecules
Classification	ROC-AUC	Physiology	BBBP	1	2039
			Tox21	12	7831
			Toxcast	617	8575
			SIDER	27	1427
			Clintox	2	1478
		Biophysics	BACE	1	1513
			MUV	17	93807
			HIV	1	41127

- The BBBP dataset is a binary classification dataset used for predicting whether a compound can penetrate the blood-brain barrier. It contains 2,039 compounds and focuses on blood-brain barrier permeability.
- The Tox21 dataset provides qualitative toxicity measurements and stress response pathways for over 10,000 compounds across 12 different in vitro assays. These assays include seven from the nuclear receptor (NR) signaling pathway and five from the stress response (SR) pathway. Each compound is labeled with binary outcomes (active/inactive) for these 12 assays, making it a multi-class classification dataset.
- The ToxCast dataset provides toxicology data for a large compound library based on in vitro high-throughput screening. It includes qualitative results from over 600 experiments for 8,576 compounds. The dataset is used for multi-class classification tasks, with each compound being evaluated across multiple toxicological endpoints.
- SIDER is a dataset that provides measurements for adverse effects of 1,427 marketed and approved drugs, categorizing drug side effects into 27 organ system classes.
- The ClinTox dataset provides data for 1,478 drug compounds with known chemical structures, encompassing two classification tasks: clinical trial toxicity and FDA approval status.

- The BACE dataset provides quantitative (IC_{50}) and qualitative (binary label) binding results for 1,513 compounds as inhibitors of human β-secretase 1 (BACE-1). It is a binary classification dataset with labels indicating whether a compound is an inhibitor
- The Maximum Unbiased Validation (MUV) dataset is a benchmark dataset for virtual screening derived from PubChem BioAssay. It contains 17 tasks with approximately 90,000 compounds, designed to validate virtual screening techniques.
- The HIV dataset is introduced by the Drug Therapeutics Program (DTP) AIDS Antiviral Screen, which tested the ability to inhibit HIV replication for over 40,000 compounds. The screening results are categorized into three classes: confirmed inactive (CI), confirmed active (CA), and confirmed moderately active (CM). However, in many applications, the latter two labels (CA and CM) are combined, making it a binary classification task between inactive (CI) and active (CA/CM).
-

1.1.2 QM9 dataset

The QM9 dataset is a quantum chemistry benchmark that includes 133,885 stable small organic molecules, each containing up to 9 heavy atoms. These molecules are a subset of the GDB-17 chemical universe, which comprises 166 billion organic molecules. Each molecule in QM9 is associated with 12 targets that cover its geometric, energetic, electronic, and thermodynamic properties, all calculated using density functional theory (DFT). Details are shown in *Supplementary Table S2.*

Supplementary Table S 2. Description of the 12 downstream tasks in the QM9 dataset.

Symbol	Meaning	Unit	Description
Alpha	Isotropic polarizability	$Bohr^3$	Measures how easily the electron cloud of a molecule can be distorted by an external electric field. A higher value indicates easier polarization.
Gap	HOMO–LUMO energy gap	eV	The energy difference between the Highest Occupied Molecular Orbital (HOMO) and the Lowest Unoccupied Molecular Orbital (LUMO). It is commonly used to assess a molecule's reactivity and stability.
HOMO	Highest Occupied Molecular Orbital	eV	Energy of the highest molecular orbital that contains electrons. A higher value indicates electrons are more easily excited or removed.
LUMO	Lowest Unoccupied Molecular Orbital	eV	Energy of the lowest molecular orbital that is not occupied by electrons. Represents the lowest accessible energy state for an electron transition.
Mu	Dipole moment	Debye	Describes the polarity of the molecule, quantifying the uneven distribution of electric charge.

Symbol	Meaning	Unit	Description
Cv	Heat capacity at constant volume	cal/mol·K	The amount of heat required to raise the temperature of a substance by one degree at constant volume. Typically calculated at 298 K.
G298	Gibbs free energy at 298 K	Hartree	The Gibbs free energy of the molecule at 298 K, used to evaluate thermodynamic stability.
H298	Enthalpy at 298 K	Hartree	Total heat content of the system at 298 K, a state function in thermodynamic calculations.
R2	Electronic spatial extent ($\langle R^2 \rangle$)	$Bohr^2$	Indicates the spatial extent of the electron cloud — how widely electrons are spread in space.
U298	Internal energy at 298 K	Hartree	The total energy of the molecule at 298 K, excluding pressure-volume work.
U0	Internal energy at 0 K	Hartree	The molecule's energy at absolute zero, representing the quantum mechanical ground-state energy.
Zpve	Zero-point vibrational energy	Hartree	The vibrational energy a molecule possesses even at absolute zero due to quantum mechanical effects.

1.2 Input feature construction

For the 1D representation, the input is an ESPF that comprises 2585 substructures.
For the 2D representation, given the SMILES string of the molecular, we used RDKit to convert it into a molecular graph representation. The input consists of atom features and bond features (details in *Supplementary Table S3)*.
For the 3D representation, the input includes atom features and position features. The atom features are the same as those used in the 2D view, while the position features refer to the spatial coordinates of the atoms.

Supplementary Table S 3. Atom and bond features.

atom features	Description
Atomic Number	[1,2....,118]+[119,120]
Chirality	Unspecified, Tetrahedral CW, Tetrahedral CCW, Other
**bond features**	**Description**
Bond Type	Single, Double, Triple, Aromatic
Bond Direction	None, End-Up-Right, End-Down-Right
Bond Stereochemistry	Stereo-None, Stereo-Z, Stereo-E, Stereo-Cis, Stereo-Trans, Stereo-Any

1.3 Experimental settings

In our implementation, pretraining on PCQM4Mv2 for 12 epochs using 2 × NVIDIA

A6000 GPUs required approximately 48 hours, with peak GPU memory usage around 20 GB per device. We set the batch size to 256 for each device and trained for 18 epochs. We utilize the Adam optimizer with a learning rate of 1e-4 to optimize the total loss, and it's weight decay is set to 1e-5. The *1D_perturbation_ratio, 2D_perturbation_ratio, 3D_perturbation_ratio* is set to 0.2, 0.25, 0.3, respectively. The important hyperparameters used to balance the two loss terms in the 3D denoising loss are set to $\lambda_2 = 1.0$ and $\lambda_{cos} = 0.1$ respectively. More about the relevant parameters of the encoding and decoding modules are given in our code.
During the fine-tuning of downstream tasks, to ensure a fair comparison, we followed the experimental setup of previous works, which involved conducting experiments using three different splits of train/val/test data. MolCL-SP is implemented in Pytorch and typically completed within 0.5–1.5 hours on a single A6000 GPU, with peak memory usage under 9 GB and are repeated three times with different seeds. More details about hyperparameters during the fine-tuning phase are provided in Supplementary Table S4.

Supplementary Table S 4. Hyperparameter and performance summary of molecular property prediction.

dataset	learning rate	weight decay	epoch	batch size	hidden size
BBBP	1e-4	1e-5	30	64	64
BACE	3e-5	1e-4	40	64	64
Clintox	1e-4	1e-5	40	128	64
SIDER	5e-4	1e-5	40	128	64
Tox21	3*1e-5	1e-2	60	64	64
Toxcast	5*1e-5	1e-5	20	128	512
HIV	1e-5	1e-5	20	64	64
MUV	1e-5	1e-4	20	64	64

1.4 The detail of all baselines.

To demonstrate the effectiveness of MolFCL in molecular property prediction, we compare it with some quality baseline models, The following is a description of them.

- MolCLR: Self-supervised graph contrastive learning method, typical pre-training-fine-tuning framework.
- GraphMVP: Self-supervised multi-view learning method that integrates 2D/3D information, pre-training followed by fine-tuning.
- Mole-BERT: Combines various self-supervised tasks (e.g., masking, contrastive), supports pre-training-fine-tuning.
- 3D InfoMax: Self-supervised 3D molecular representation learning method, commonly used in pre-training-fine-tuning pipeline.

- MOLEBLEND: Multimodal self-supervised learning framework, combining different perspectives for molecular representation, used for fine-tuning.
- MoleculeSDE: Based on stochastic differential equations (SDEs), self-supervised molecular modeling method, supports pre-training-fine-tuning.
- Distance Prediction: A typical self-supervised task (predicting interatomic distances), commonly used for GNN pre-training.
- 3D InfoGraph: Graph-level contrastive learning based on 3D structures, extended from InfoGraph, self-supervised + fine-tuning.
- GROVER: A pre-trained model that integrates domain knowledge to enhance molecular representation learning, commonly used for fine-tuning on downstream tasks such as property prediction and drug discovery.
- GraphMAE: A self-supervised model based on masked autoencoders for pre-training molecular graph representations, often used as a pre-training framework to generate embeddings for downstream tasks.

1.5 Calculation process of Transformer, GIN and Dimnet

1.5.1 Transformer

The Transformer architecture primarily consists of two key components: a multi-head attention layer and a feed-forward network. The multi-head attention layer is composed of parallel self-attention mechanisms that compute scaled dot-product attention. This allows the model to focus on different subspaces of information within the input substructures. Let h_n^d represent the input to the n-th block, where $h_0^d = H_{input}^{1d}$. The multi-head attention mechanism operates with u heads as follows:

$$\begin{aligned} head_i^{1d} &= softmax\left(\frac{Q_n^{1d} K_n^{1d}}{\sqrt{d}}\right) V_n^{1d} \\ &= softmax\left(\frac{h_n^{1d} W_n^Q (h_n^{1d} W_n^K)^T}{\sqrt{d}}\right) h_n^{1d} W_n^V, \end{aligned}$$

$$MultiHead(h_n^{1d}) = concat\,(head_1^{1d}, \ldots, head_u^{1d}) W_n^T,$$

Here, W_n^Q, W_n^K, W_n^V, and W_n^T are the weight parameters, and $\frac{1}{\sqrt{d}}$ is a scaling factor that depends on the dimension of each attention head. Following the multi-head attention mechanism, a feed-forward layer is applied to enhance the model's reasoning capabilities.

$$H_m^{1d} = f(MultiHead\,(h_n^{1d}) W_n^{f,1} + b_n^{f,1}) W_n^{f,2} + b_n^{f,2},$$

Here, $W_n^{f,1}$, $W_n^{f,2}$, $b_{f,n}^1$, and $b_{f,n}^2$ are the weight parameters, and H_m^{1d} is the output of the n -th Transformer encoder block.

1.5.2 GIN

The propagation rule for the lth layer in the Graph Isomorphism Network (GIN) is given by:

$$h_a^{2d,l+1} = \text{MLP}\,(h_a^{2d,l} + \sum_{b\in\mathcal{N}(a)} (h_b^{2d,l} + \text{MLP}\,(h_{ab}^{2d,l}))),$$

where $h_a^{2d,l}$ and $h_{ab}^{2d,l}$ are the 2D atom representation and the neighboring bond representation at the l-th layer, respectively, and MLP denotes a multi-layer perceptron.

1.5.3 Dimnet

DimeNet is a method used in graph neural networks (GNNs) specifically designed to handle molecular graphs and extract complex structural information from them. Its core idea is to combine continuous distance encoding and directional information with traditional GNN structures to better capture the spatial relationships between atoms in a molecule.

Each atomic node is initialized with an embedding representation of its type, or it can be initialized with other features (e.g., atomic charge, size). Edge features are then obtained by calculating geometric distances (e.g., Euclidean distances) between pairs of atoms. These distances can be used directly as edge features or further processed by distance conversion functions, e.g.:

$$r_{ij} = \| r_i - r_j \|$$

where r_i and r_j are the coordinates of atoms i and j, respectively.

DimeNet incorporates both position and directional information. The distance between atoms is encoded as continuous edge features using a smooth function (e.g., a Gaussian function):

$$\emptyset_{ij} = \exp\,(-\frac{r_{ij}^2}{\sigma^2})$$

where r_{ij} is the distance between atoms i and j, and σ is the width parameter.

Directional information is also encoded in a similar way to capture the relative direction between atoms.

Each atomic node is updated using graph convolution, considering the information from its neighbors. DimeNet uses a convolution operation with edge features:

$$h_i^{(k+1)} = Aggregate_j(f(h_i^{(k)}, h_j^{(k)}, \emptyset_{ij}))$$

where $h_i^{(k)}$ is the representation of atom i at the k-th layer, $\emptyset_{ij}$ is the edge feature between atoms i and j, and $Aggregate_j$ represents the aggregation operation over neighboring nodes.

The representations of all atomic nodes are aggregated to obtain a final embedding for the entire molecule. This embedding can then be used for downstream tasks (such as regression, classification, etc.).

Finally, DimeNet outputs the molecular representation, which can be used for various tasks, such as predicting molecular properties or reactivity.

1.6 Non-overlapping substructure perturbations strategy

For the convenience of readers' understanding, the following is a pseudocode for us to implement the atomic substructure on its part. The specific content is as follows:

```
FOR each atom_idx in [0 .. atom_count - 1] DO
    SET flag = FALSE
    SET current_token_pos = temp_token_pos

    FOR each token in tokenized_smiles starting from current_token_pos DO
        IF current_match_atom_cnt[temp_token_pos] >= match_atoms_cnt[temp_token_pos] THEN
                INCREMENT temp_token_pos by 1
            ELSE
                SET token_atoms = match_atoms[temp_token_pos]
                IF mol_atoms[atom_idx] in token_atoms THEN
                    atom_substructure_mapping[atom_idx] = temp_token_pos
                    INCREMENT current_match_atom_cnt[temp_token_pos] by 1
                    REMOVE mol_atoms[atom_idx] from token_atoms
                    SET flag = TRUE
                    BREAK inner loop
                END IF
                INCREMENT temp_token_pos by 1
            END IF
    END FOR

    IF flag == FALSE THEN
        SET temp_token_pos = current_token_pos
    END IF
END FOR
```

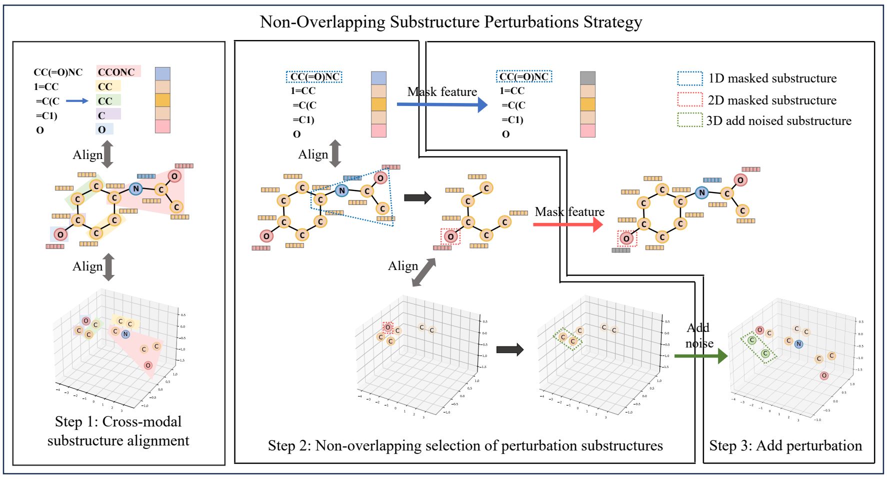

Supplementary Fig. S 1. Illustrative example of the Non-overlapping substructure perturbations strategy.

2 Additional experiments and analysis

2.1 More visualization findings on molecular representations

To further demonstrate the effectiveness of the learned molecular representations, we conducted t-SNE visualization and molecular retrieval on the BBBP dataset. As shown in Supplementary Fig. S2, the results highlight the informativeness of our representations and the efficacy of the pretraining strategy.

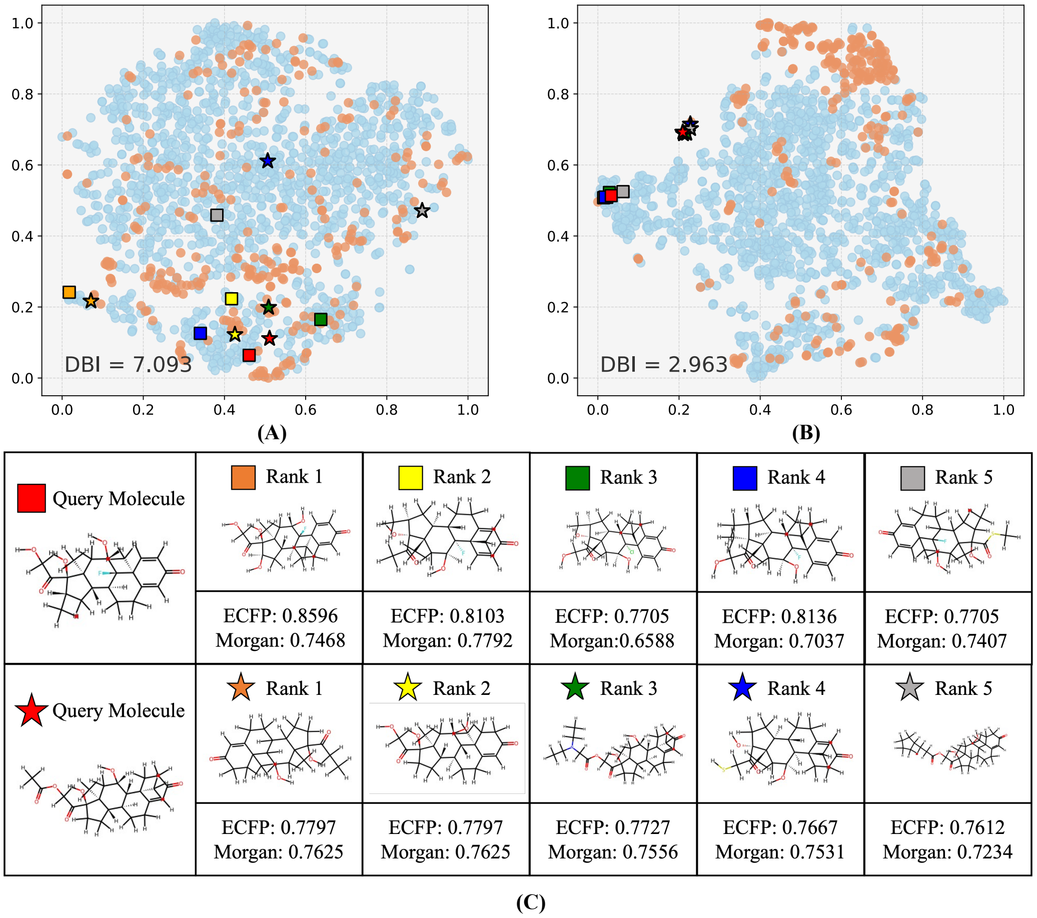

Supplementary Fig. S 2. Visualization of molecular representations. (A, B) t-SNE plots of BBBP dataset representations extracted by MolCL-SP without and with pretraining. (C) Molecular retrieval results.

2.2 Profile of Predefined Functional Groups

Below are the 48 functional groups that we predefined in the model interpretability exploration experiments.

Supplementary Table S 5. Predefined 48 functional groups in interpretable experiments and their introduction.

Functional Group	Chemical Formula	Description
Hydroxyl	-OH	An alcohol group with a hydroxyl (-OH) attached to a carbon.
Ether	R-O-R’	An oxygen atom connected to two carbon atoms (R-O-R’).
Primary Amine	$R-NH_2$	An amine group with two hydrogen atoms attached to a nitrogen ($R-NH_2$).
Tertiary Amine	R-NR’-R”	An amine group with three carbon atoms attached to a nitrogen (R-NR’-R”).
Alkyl	$R-CH_3$	A saturated carbon atom with four single bonds ($R-CH_3$).
Carbonyl	C=O	A carbon atom double-bonded to an oxygen (C=O).
Carboxamide	$R-CO-NH_2$	A carbonyl group attached to an amine ($R-CO-NH_2$).
Chloro	R-Cl	A chlorine atom attached to a carbon (R-Cl).
Sulfide	R-S-R’	A sulfur atom connected to two carbon atoms (R-S-R’).
Alkenyl	C=C	A carbon-carbon double bond.
Carboxyl	R-COOH	A carboxylic acid group with a carbonyl and a hydroxyl.
Carboalkoxy	R-COOR’	An ester group with a carbonyl and an ether.
Halo	R-X (X=F, Cl, Br, I)	A halogen atom attached to a carbon.
Phenyl	C_6H_5-	A benzene ring without any additional substituents.
Pyridyl	C_5H_5N	A pyridine ring with a nitrogen atom in the ring.
Secondary Amine	R-NHR’	An amine group with one hydrogen and one carbon attached to a nitrogen.
Bromoalkane	R-Br	A bromine atom attached to a carbon.
Nitrile	R-CN	A carbon atom triple-bonded to a nitrogen.
Fluoro	R-F	A fluorine atom attached to a carbon.
Imide	$R-CONHNH_2$	A cyclic compound with a carbonyl and two nitrogen

Functional Group	Chemical Formula	Description
		atoms.
Secondary Aldimine	R-C=N-R’	An imine group with a carbon-carbon double bond.
Secondary Ketmine	R-C=NC=O	An imine group with a carbonyl.
Nitro	$R-NO_2$	A nitro group with a nitrogen atom double-bonded to two oxygen atoms.
Aldehyde	R-CHO	A carbonyl group at the end of a carbon chain.
Alcohol	-OH	An alcohol group with a hydroxyl attached to a carbon.
Acid Chloride	R-COCl	A carbonyl group attached to a chlorine.
Amide	$R-CO-NH_2$	A carbonyl group attached to an amine.
Anhydride	R-CO-O-CO-R’	An acid anhydride with two carbonyl groups connected by an ether.
Thiol	-SH	A sulfur atom with a hydrogen attached.
Isocyanate	R-N=C=O	A nitrogen atom double-bonded to a carbonyl.
Isothiocyanate	R-N=C=S	A nitrogen atom double-bonded to a thiocarbonyl.
Benzyl	$C_6H_5-CH_2-$	A benzyl group with a benzene ring attached to a carbon.
Acetal	R-C(O)(O-R’)-	A geminal diether with two ether groups attached to the same carbon.
Ketone	R-CO-R’	A carbonyl group with two carbon atoms attached.
Urethane	$R-O-CO-NH_2$	An ester of carbamic acid.
Azo	R-N=N-R’	A nitrogen-nitrogen double bond.
Triazole	$C_2H_3N_3$	A five-membered aromatic ring with three nitrogen atoms.
Pyrrole	C_4H_4NH	A five-membered aromatic ring with one nitrogen atom.
Furan	C_4H_4O	A five-membered aromatic ring with one oxygen atom.
Thiophene	C_4H_4S	A five-membered aromatic ring with one sulfur atom.
Pyridine	C_5H_5N	A six-membered aromatic ring with one nitrogen atom.
Thioether	R-S-R’	A sulfur atom connected to two carbon atoms.
Alkene	C=C	A carbon-carbon double bond.
Alkyne	C≡C	A carbon-carbon triple bond.
Benzene	C_6H_6	A benzene ring.

Functional Group	Chemical Formula	Description
Aromatic	C_6H_6	An aromatic ring structure.
Sulfonyl	$R\text{-}SO_2\text{-}R'$	A sulfur atom double-bonded to an oxygen and attached to a carbonyl.
Benzyl Alcohol	$C_6H_5\text{-}CH_2OH$	A benzyl group with an alcohol attached to the benzene ring.

2.3 Ablation study

2.3.1 Module ablation research

In the “w/o non-overlapping” variant, substructure perturbations are applied by randomly selecting substructures without enforcing any non-overlapping constraints. As a result, multiple perturbed substructures may share overlapping atoms or bonds, leading to redundancy.

To quantify this effect, we computed the overlap rate defined as the proportion of atoms that appear in more than one perturbed substructure. The overlap rate in the “w/o non-overlapping” variant is approximately 48%, indicating a high degree of redundancy. In contrast, our full MolCL-SP model employing the non-overlapping perturbation strategy achieves a much lower overlap rate of about 15%.

This significant difference highlights the importance of the non-overlapping design in reducing redundancy and improving the quality of contrastive augmentations.

Supplementary Table S 6. Results of ablation experiments of MolCL-SP and its four variants on eight downstream task datasets of MoleNet.

Dataset	MolCL-SP	w/o 1D	w/o 2D	w/o 3D	w/o non-overlapping
BBBP	**74.40 ± 0.7**	73.3 ± 0.5	73.0 ± 0.7	72.1 ± 0.8	<u>73.4 ± 0.8</u>
Tox21	**76.9± 1.0**	74.5 ± 0.7	73.0 ± 0.7	74.8 ± 1.1	<u>75.2 ± 1.1</u>
ToxCast	**68.4 ± 0.2**	67.2 ± 0.3	67.2 ± 0.5	66.8 ± 0.3	<u>67.6 ± 0.5</u>
SIDER	**65.6 ± 0.1**	64.1 ± 0.3	63.6 ± 0.2	64.3 ± 0.1	<u>64.4 ± 0.4</u>
ClinTox	**99.4 ± 0.2**	97.9 ± 0.7	98.6 ± 0.7	<u>98.9 ± 0.5</u>	98.1 ± 0.2
MUV	**80.2 ± 0.8**	78.1 ± 0.8	78.0 ± 1.0	<u>79.2 ± 1.2</u>	78.8 ± 0.9
HIV	**80.9 ± 0.5**	77.0 ± 0.4	78.2 ± 0.1	<u>79.2 ± 0.6</u>	78.2 ± 0.6
BACE	**82.9 ± 1.0**	<u>81.3 ± 1.4</u>	80.1 ± 1.2	81.1 ± 0.6	80.9 ± 1.0

The best result is shown in bold and the second best result is underlined.

2.3.2 Component ablation research

To further assess the impact of encoder selection on MolCL-SP’s performance, we conducted ablation experiments by replacing the encoders for the 1D, 2D, and 3D modalities with alternative architectures, and evaluated the results on the MoleculeNet

and QM9 datasets. Specifically, for the1D modality, we compared the Transformer with LSTM and CNN (all with 128-dimensional outputs); for the 2D modality, we compared GIN with GCN and GraphSAGE; and for the 3D modalit, we compared DimeNet with SchNet and Pafnucy.

The results show that replacing any modality's encoder led to a performance drop. The first row of the table shows the encoder configuration used by Mol-SP. For instance, replacing the 1D encoder with LSTM reduced the average AUC by 2.3% (with the largest drop of 3.1% on the MUV dataset), due to LSTM's weaker capability in modeling long-range substructure dependencies compared to the Transformer's self-attention mechanism. In the 2D modality, replacing GIN with GCN decreased the AUC by 1.8% on tasks requiring the discrimination of isomorphic molecules (e.g., BBBP), confirming GIN's advantage in graph isomorphism recognition. For the 3D modality, replacing DimeNet with SchNet increased the mean absolute error (MAE) of dipole moment (μ) prediction on QM9 by 0.05, as SchNet models only distance features, whereas DimeNet's angle-aware design better captures spatial interactions. Notably, replacing encoders in two or more modalities resulted in an accumulative performance decline (average AUC drop of 4.7%), indicating that the original encoder combination was optimized for cross-modal feature complementarity. These results confirm the rationale of selecting modality-specific encoders—Transformer for contextual modeling of 1D sequences, GIN for topological features of 2D graphs, and DimeNet for spatial interactions in 3D structures—whose synergy forms the foundation of MolCL-SP's performance.

Supplementary Table S 7. The ablation experimental results of MolCL-SP and its variants formed by replacing encoders in various modalities in 2D and 3D fine-tuning tasks.

1D encoder	2D encoder	3D encoder	Avg AUC of MoleculeNet	Avg MAE of QM9
Transformer	GIN	DimeNet	**78.84 ± 0.52**	**10.353 ±0.12**
LSTM	GIN	DimeNet	76.51 ± 0.63	10.421 ± 0.15
CNN	GIN	DimeNet	77.03 ± 0.58	<u>10.392±0.13</u>
Transformer	GCN	DimeNet	77.04 ± 0.61	10.405 ±0.14
Transformer	GraphSAGE	DimeNet	76.82 ± 0.59	10.418 ± 0.16
Transformer	GIN	SchNet	<u>77.26 ± 0.55</u>	10.408 ± 0.13
Transformer	GIN	Pafnucy	76.95 ± 0.60	10.432 ± 0.17

The best result is shown in bold and the second best result is underlined.

2.4 Parameters sensitivity analysis

To validate the robustness of the denoising loss weights $(\lambda_{L2}\ and\ \lambda_{cos})$ in Equation 6, we conducted a sensitivity analysis by varying their values during pretraining and evaluating downstream performance on the BBBP and BACE datasets (from MoleculeNet) using ROC-AUC as the metric . The experimental setup involved testing combinations of $\lambda_{L2} \in \{0.1, 1, 10\}$ with $\lambda_{cos} \in \{0.1, 1, 10\}$ during pretraining on the PCQM4Mv2 dataset, while keeping other hyperparameters unchanged . Each configuration was run three times with different random seeds, and results are summarized in Fig S3 and Fig S4.
The results indicate that the model maintains stable performance across reasonable weight ranges, with fluctuations in ROC-AUC within 2% for both datasets, confirming the robustness of our method to the choice of λ_{L2} and λ_{cos}. Notably, the optimal performance at $\lambda_{L2} = 1.0$ and $\lambda_{cos} = 0.1$ on both fine-tuning task aligns with the original parameter selection, suggesting that prioritizing the L2 loss (which directly penalizes coordinate errors) while retaining a small contribution from the cosine similarity loss (which preserves directional information) is beneficial for learning meaningful 3D molecular representations. This balance likely enhances the model's ability to capture both geometric precision and structural relationships, which are critical for downstream property prediction tasks .

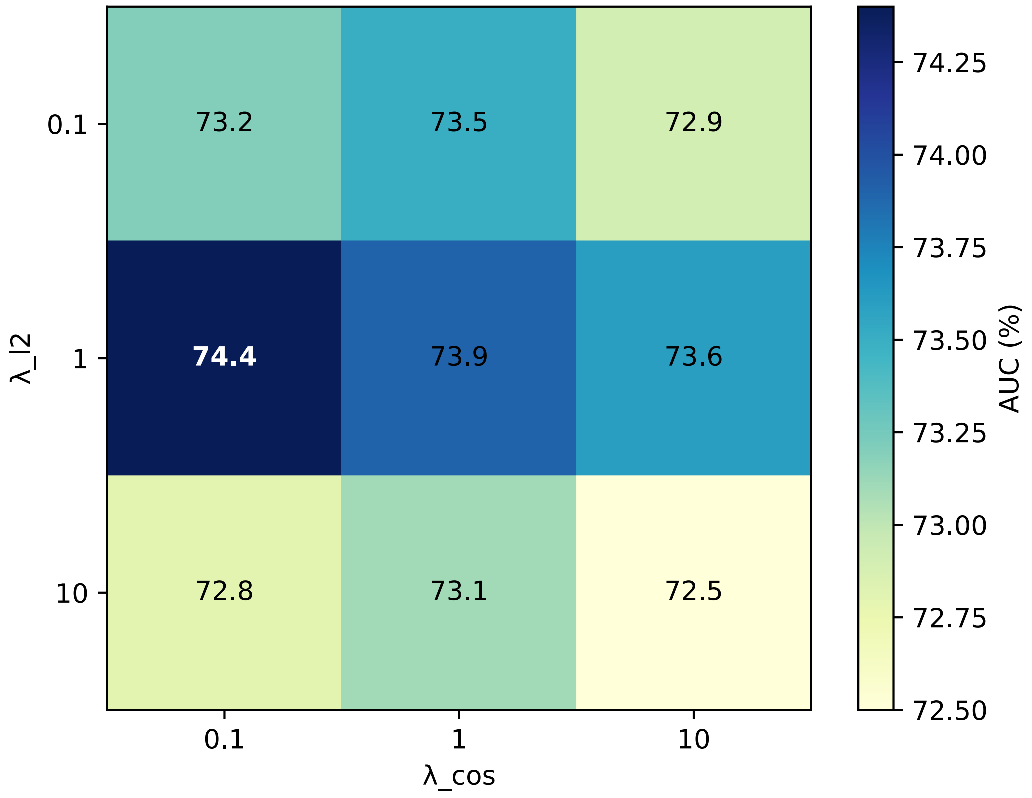

Supplementary Fig. S 3. Parameter sensitivity heatmap on BBBP dataset.

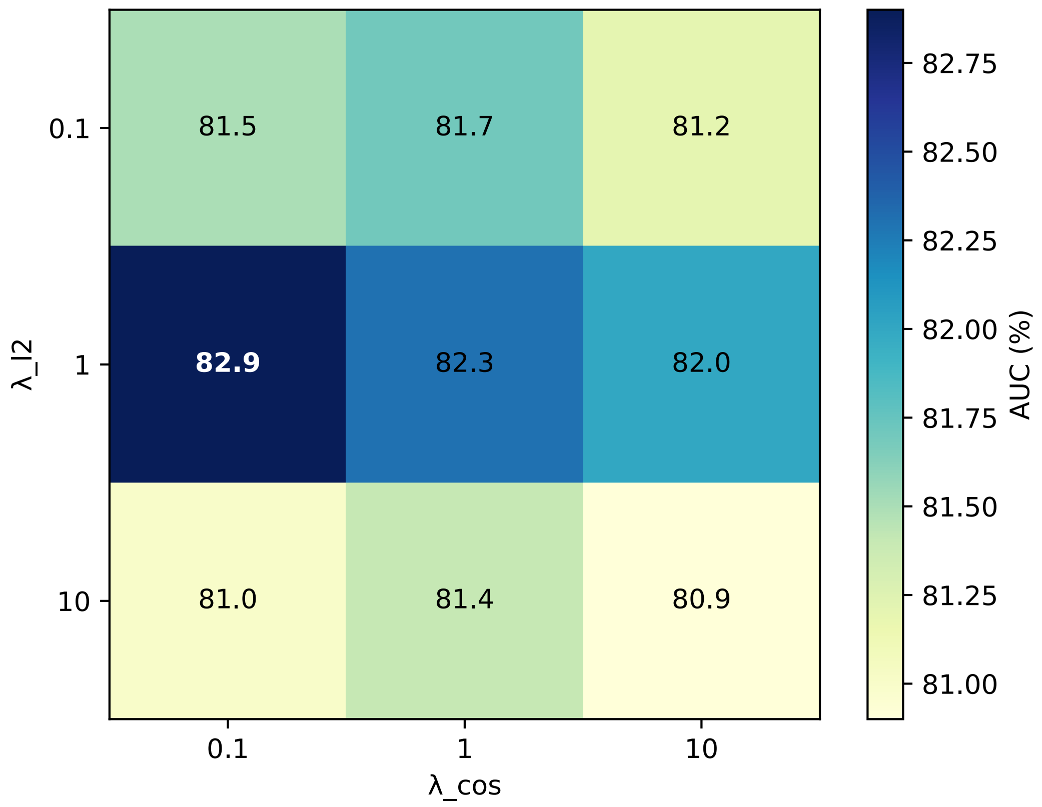

Supplementary Fig. S 4. Parameter sensitivity heatmap on BACE dataset.

2.5 Statistical investigation of interpretability

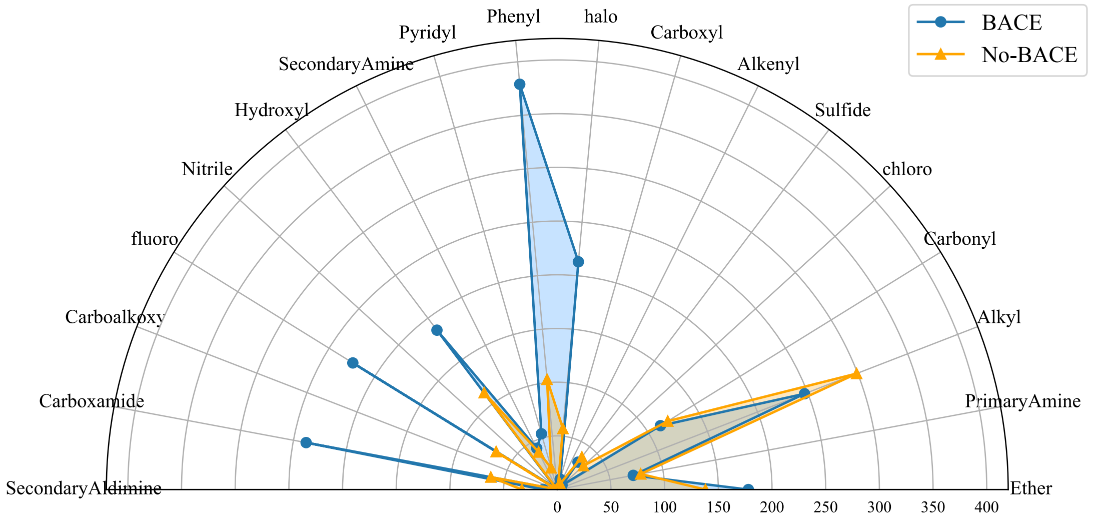

Supplementary Fig. S 5. Statistical investigation of interpretability for the functional group prompt: analysis on the BACE dataset.

2.6 Performance of MolCL-SP on the drug-drug interaction prediction task

2.6.1 Drug-drug interaction (DDI) prediction task

Under this task settings, interaction types were one-hot encoded and concatenated with the embeddings of the two drugs, then passed through a multilayer perceptron to predict the interaction probability.

Under the transductive setting, all triplets in the dataset were split into training, validation, and test sets with a 6:2:2 ratio. To ensure robustness, this procedure was repeated three times to create three stratified random folds. Methods were evaluated using the same splits for fair comparison.

In the inductive setting, 20\% of drugs in the DrugBank dataset were randomly selected as new (unseen) drugs, and all interactions involving these drugs were excluded from training. The remaining drugs were considered known. To evaluate inductive performance, we adopted two partition strategies:

- **P1 Partition (new drug, new drug):** the test set contains DDIs exclusively between novel drugs;
- **P2 Partition (new drug, existing drug):** each test DDI involves one known and one novel drug.

All non-test data were used for training. To ensure robustness, we conducted three independent experiments for each scenario.

Supplementary Table S 8. Performance assessment of MolCL-SP and comparison baselines under transductive setting on DrugBank dataset.

Method	ACC	AUC	AP	F1
Morgan	90.78 ± 0.36	92.51 ± 0.21	92.87 ± 0.22	91.68 ± 0.75
GMPNN-CS	95.30 ± 0.05	98.46 ± 0.01	97.94 ± 0.02	95.39 ± 0.05
DGNN-DDI	96.09 ± 0.27	98.94 ± 0.31	98.51 ± 0.37	96.16 ± 0.31
MSAN	<u>96.69 ± 0.61</u>	<u>99.27 ± 0.78</u>	<u>99.12 ± 1.00</u>	<u>97.04 ± 0.60</u>
SSI-DDI	96.33 ± 0.09	98.95 ± 0.08	98.57 ± 0.14	96.38 ± 0.09
MolCL-SP	**97.65 ± 0.04**	**99.34 ± 0.16**	**99.27 ± 0.07**	**96.48 ± 0.04**

The best result is shown in bold and the second best result is underlined.

Unlike methods relying on global molecular encoding (e.g., Morgan fingerprints or holistic graph embeddings), MolCL-SP's non-overlapping substructure perturbations direct the model to focus on modular components (e.g., functional groups, aromatic rings) rather than entire scaffolds. This encourages learning patterns that transcend specific molecular architectures: hydroxyl groups (-OH) retain their role in solubility across diverse scaffolds, and carboxyl groups (-COOH) consistently drive binding with basic amino acids, regardless of the global structure. By prioritizing these local, recurring features, the model avoids over-reliance on dataset-specific scaffolds and instead captures universally applicable chemical rules. In essence, substructure-level perturbations enable MolCL-SP to learn a "chemical grammar" grounded in local

patterns, allowing it to interpret novel drugs as combinations of familiar, functionally meaningful components. This reduces dependence on global scaffolds, directly enhancing inductive generalization—critical for evaluating uncharacterized compounds in drug discovery.

To further assess the impact of fine-tuning on MolCL-SP's performance in the Drug–Drug Interaction (DDI) prediction task, we compared two settings under the inductive evaluation on the DrugBank dataset. In the frozen setting, embeddings were directly extracted from the pretrained MolCL-SP encoders and Transformer fusion module, and kept fixed when training the classification MLP. In the fine-tuning setting, all parameters of the pretrained MolCL-SP (encoders and fusion module) were updated jointly with the MLP during training.

Supplementary Table S 9. Performance comparison between frozen and fine-tuned MolCL-SP on DrugBank dataset(inductive setting).

Setting	Partition	ACC(%)	AUC(%)	AP (%)	F1 (%)
Frozen	P1	72.03 ± 0.42	80.15 ± 0.67	80.42 ± 0.55	71.26±0.48
Fine-tuned	P1	74.25 ± 0.21	82.56 ± 0.57	83.11 ± 0.96	73.04±0.47
Frozen	P2	80.21 ± 0.63	88.06 ± 0.79	87.94 ± 0.72	76.31 ± 0.61
Fine-tuned	P2	82.57 ± 0.81	90.48± 1.05	90.09 ± 0.93	78.84 ± 0.98

Table S9 reports the performance comparison. Across both partitions, fine-tuning consistently improved results, achieving 2.41% AUC improvement in P1 and 2.42% AUC improvement in P2 compared to the frozen setting. The improvements were more pronounced in P1, indicating that fine-tuning is especially beneficial for predicting interactions between entirely novel drugs. These findings demonstrate that while MolCL-SP's pretrained embeddings already provide strong molecular representations, task-specific adaptation further enhances predictive capability.

2.6.2 Drug–disease association (DDA) prediction task

To further evaluate the generalizability of MolCL-SP in the drug–disease association (DDA) prediction task, we applied it to identify potential therapeutic relationships between drugs and diseases. DDA prediction aims to uncover the therapeutic potential of existing drugs for diseases that have not yet been reported, requiring the model to simultaneously capture the chemical features of drugs, the pathological mechanisms of diseases, and their latent association patterns. This task serves as an important scenario for assessing the cross-task transferability of molecular representations.

We constructed a comprehensive DDA dataset by integrating information from three authoritative sources: (1) DrugBank (Wishart et al., 2018), containing 1,652 FDA-approved drugs and their known indications; (2) DisGeNET, comprising 1,200 diseases and their associated gene phenotypes; and (3)PharmGKB, providing clinical drug–disease association data. The final dataset contains 28,743 known effective

associations (positive samples) and an equal number of randomly sampled negative samples, screened to exclude potential false negatives. Drug representations were generated using1D SMILES, 2D molecular graphs, and 3D conformations (consistent with the main framework), while disease features were obtained by integrating phenotypic descriptions from OMIM and disease-related gene sets from GeneCards, encoded into 128-dimensional embeddings via a BERT model.

The MolCL-SP-based DDA prediction pipeline is designed as follows: first, pretrained MolCL-SP encoders generate 128-dimensional multimodal fused embeddings for drugs, preserving their substructural, topological, and spatial features; second, a bidirectional attention module models the interactions between drug and disease embeddings, focusing on associations between functional substructures of drugs and key disease gene pathways; finally, the interaction features are fed into a two-layer MLP classifier, trained with binary cross-entropy loss, to predict the probability of drug–disease associations.

2.7 Computational Efficiency Analysis

All experiments were conducted on a workstation equipped with an NVIDIA RTX A6000 GPU and 64GB RAM. To evaluate the computational efficiency of MolCL-SP, we measured training time per epoch, average inference latency per sample, and GPU memory consumption, comparing against the lightweight state-of-the-art model Mole-BERT under identical conditions.

Training time: MolCL-SP requires approximately 150 minutes per epoch, which is about 6 times longer than Mole-BERT's 25 minutes per epoch.

Inference time: The average inference latency per sample for MolCL-SP is around 10 milliseconds, roughly twice that of Mole-BERT (5 milliseconds).

GPU memory usage: MolCL-SP consumes about 20GB of GPU memory during training, approximately 3.8 times the 5.2GB used by Mole-BERT.

Despite the increased training time and memory demands, the inference latency of 10 milliseconds per sample remains within an acceptable range for practical drug screening scenarios. Leveraging commonly used distributed and parallel computing techniques in large-scale screening pipelines, MolCL-SP is capable of efficiently processing millions of compounds within reasonable time frames.

Therefore, we conclude that MolCL-SP is indeed feasible for large-scale drug screening scenarios, particularly when combined with appropriate hardware infrastructure and software optimizations.
